# Supplementary figures and images for: SARS-CoV-2 infection in the Syrian hamster model causes inflammation as well as type I interferon dysregulation in both respiratory and non-respiratory tissues including the heart and kidney
Source: PLoS Pathog. 2021 Jul 15;17(7):e1009705. doi: 10.1371/journal.ppat.1009705 (PMC8282065; doi:10.1371/journal.ppat.1009705)

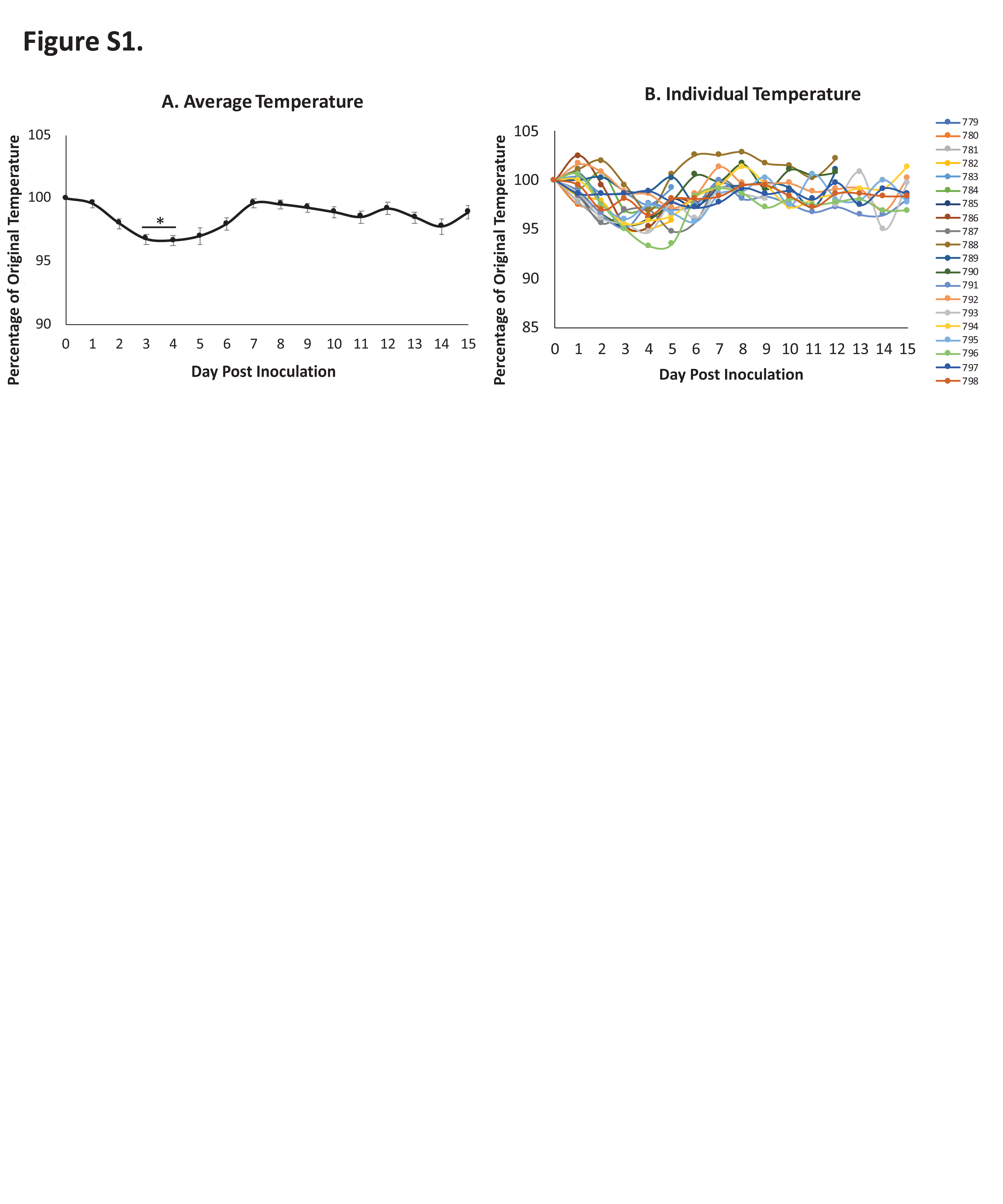

Supplement: S1 Fig — Syrian hamsters were intranasally inoculated with the severe acute respiratory syndrome coronavirus 2 (SARS-CoV-2) at 105 TCID50. Temperature (A and B) was recorded for 15 days post inoculation. Average results (A) show the mean. Individual results represent each animal (B). * indicates a p-value less than 0.05 determined by ANOVA comparing hamsters on the days post inoculation to baseline (day 0). Error bars indicate +/- standard error (SE). (TIF) [file ppat.1009705.s001.tif]

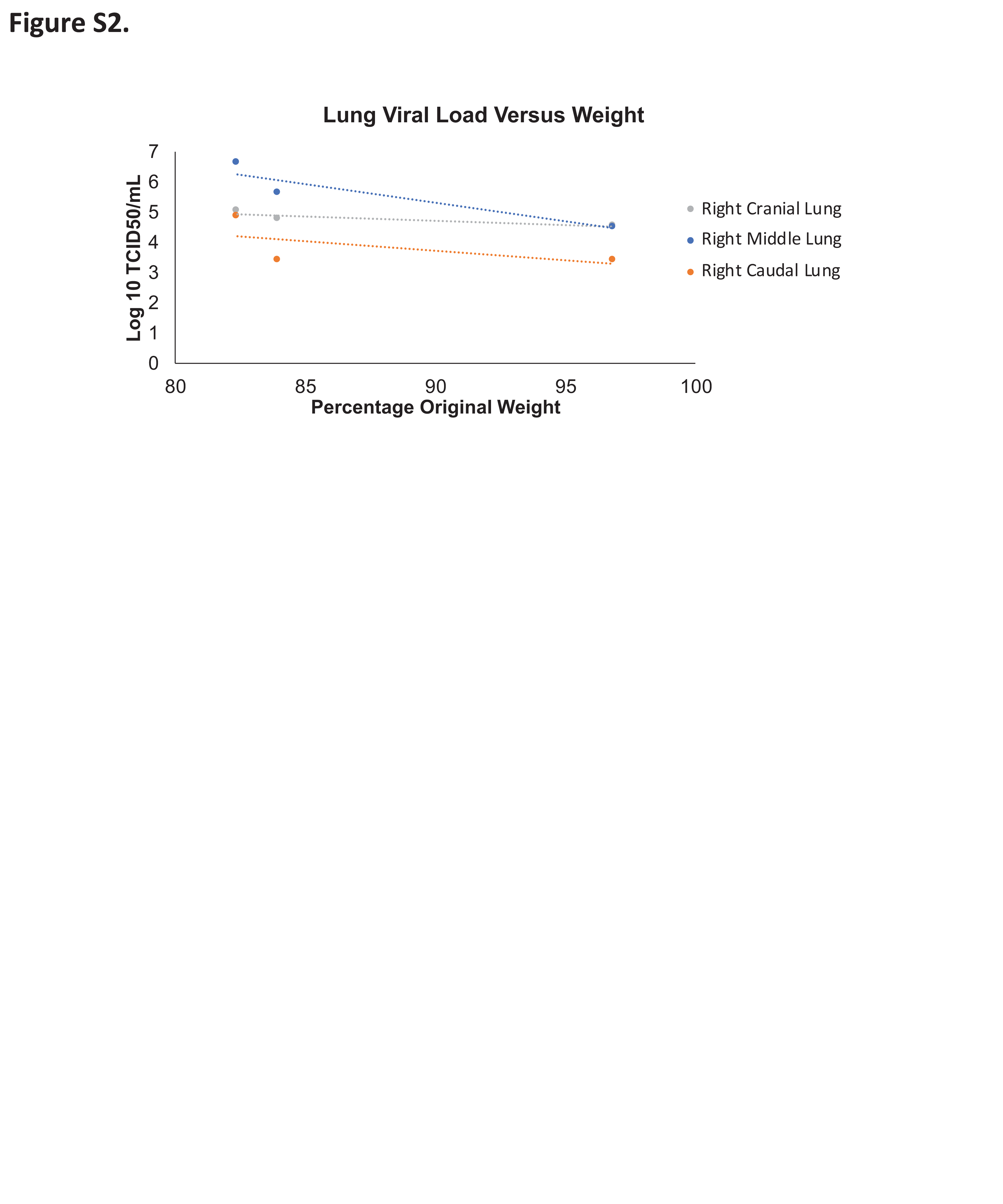

Supplement: S2 Fig — Live viral titer in the right cranial, right middle and right caudal lung lobes negatively correlated with weight change on day 5 post infection. Right cranial lung: R-value = -0.91 (p-value = 0.27); right middle lung: R-value = -0.92 (p-value = 0.25); right caudal lung: R-value = -0.58 (p-value = 0.61). (TIF) [file ppat.1009705.s002.tif]

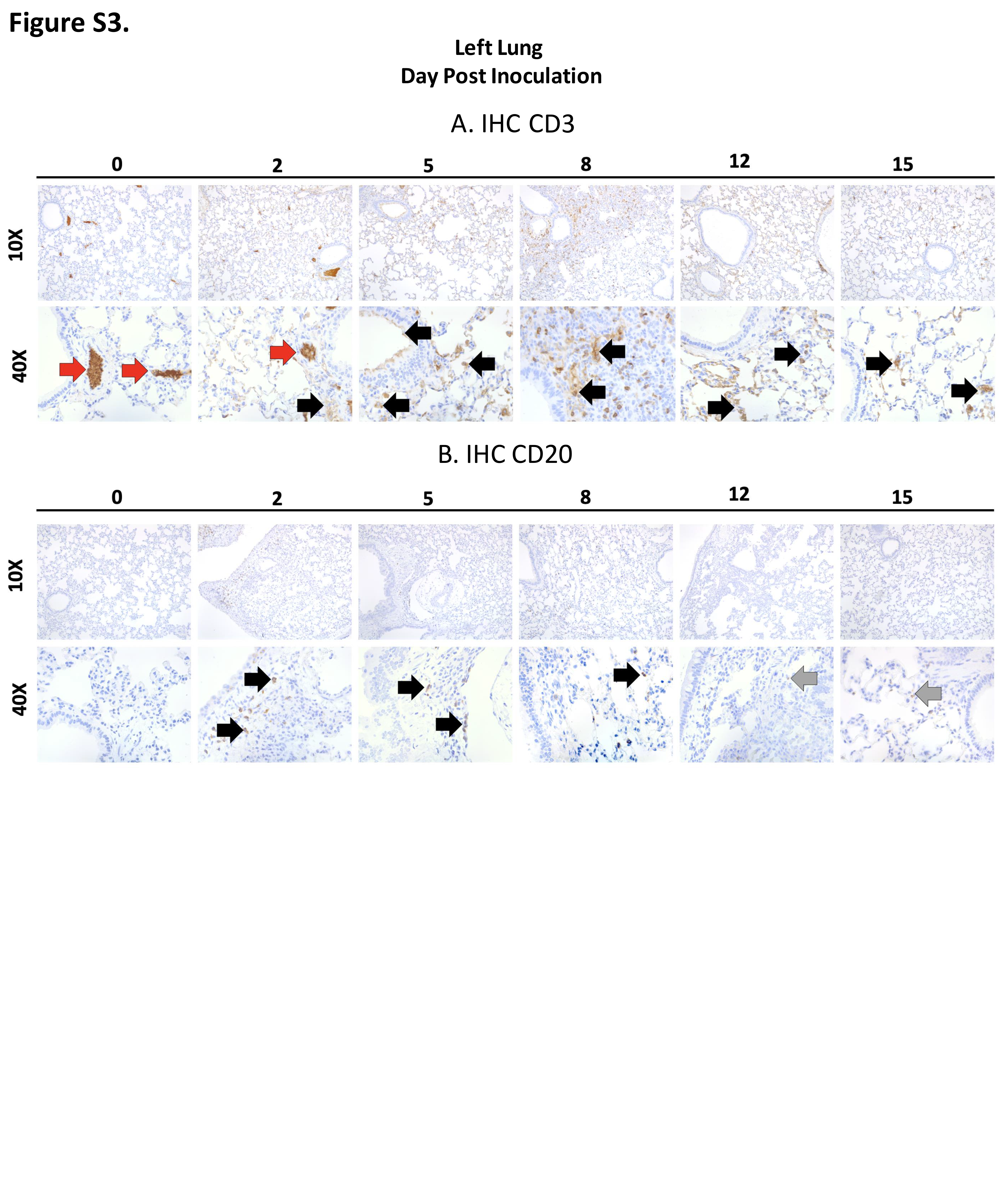

Supplement: S3 Fig — The left lung was collected from each animal at necropsy and perfused with 10% formalin prior to paraffin block embedding. The lung was stained for the presence of CD3 as a marker of T cells (A) or CD20 as a marker of B cells (B). Red arrows indicate high amounts of antigen staining, black arrows indicate intermediate amounts of antigen staining, grey arrows indicate low amount of antigen staining by IHC using target specific antibodies. Stained tissues were visualized and imaged using the Leica DMI100 Brightfield microscope and DMC5400 20 MP color CMOS camera. Images were captured and 10X and 40X. Images shown are representative of 3 animals per group, per timepoint. (TIF) [file ppat.1009705.s003.tif]

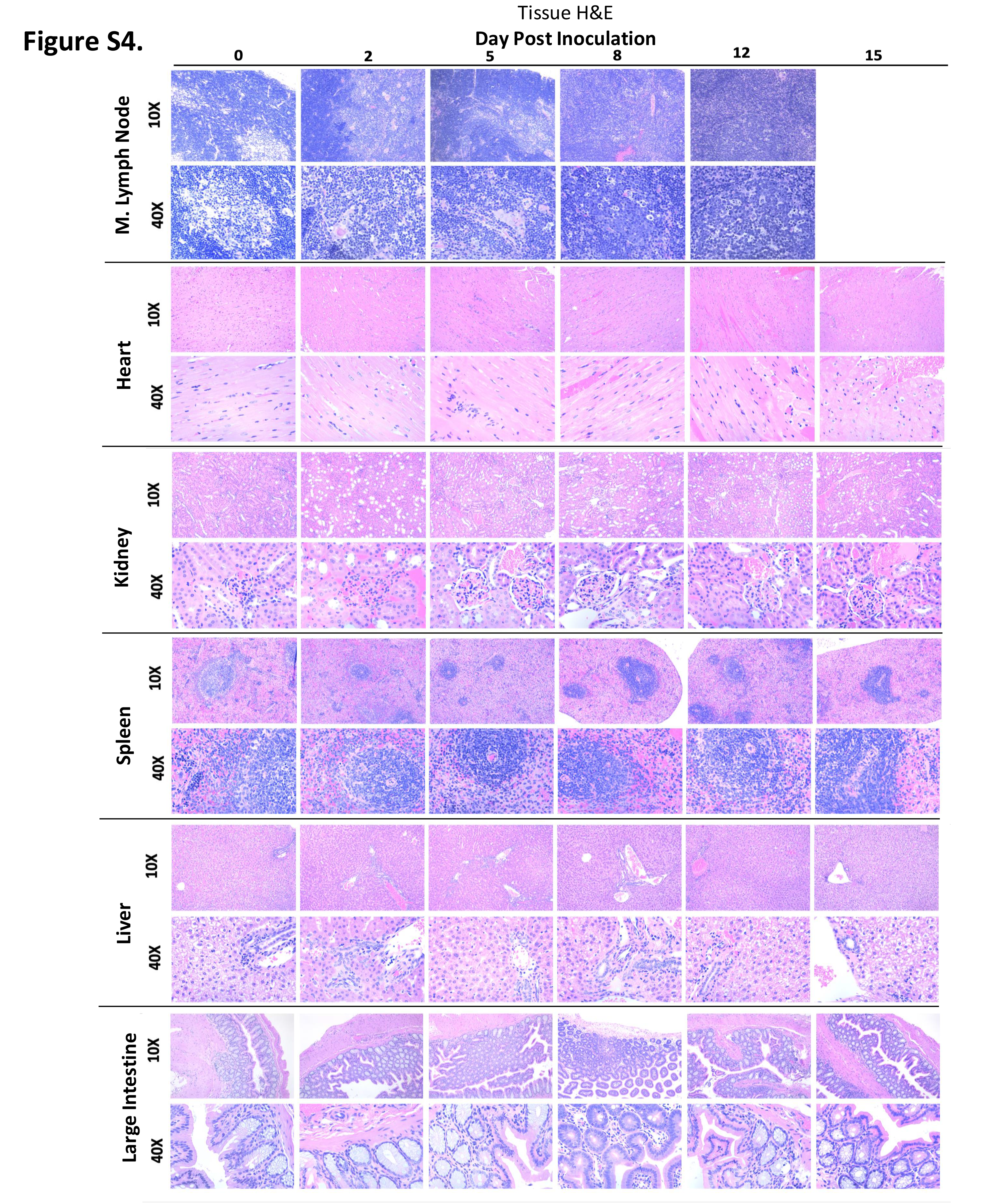

Supplement: S4 Fig — The mediastinal (M) lymph node, heart, kidney, spleen, liver and large intestine were collected from each animal at necropsy and fixed in 10% formalin prior to paraffin block embedding. All tissues were analyzed by H&E. Stained tissues were visualized and imaged using the Leica DMI100 Brightfield microscope and DMC5400 20 MP color CMOS camera. Images were captured and 10X and 40X. Images shown are representative of 3 animals per group, per timepoint. (TIF) [file ppat.1009705.s004.tif]

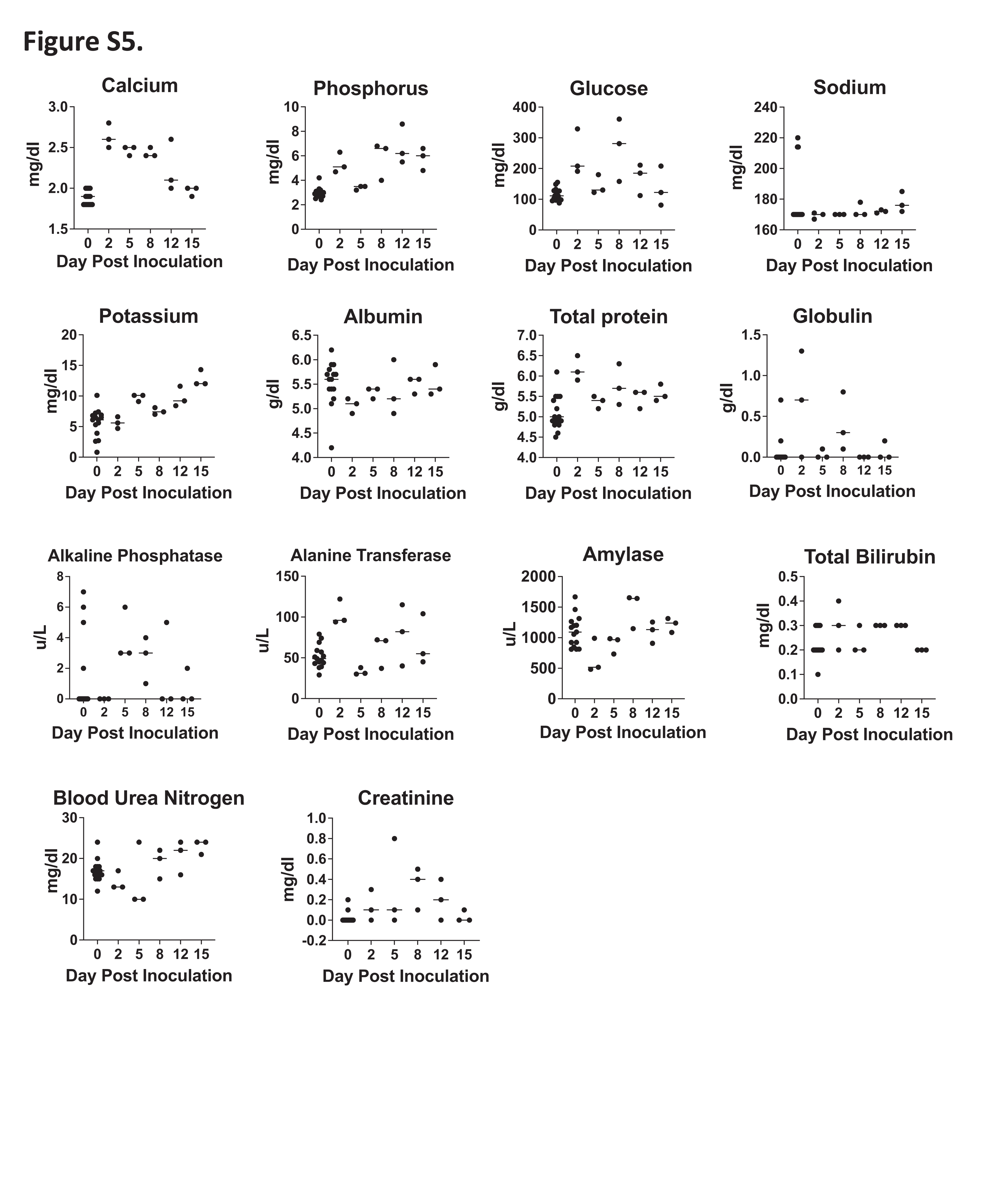

Supplement: S5 Fig — Plasma was separated from blood collected throughout the time course. Plasma was then assessed for 14 molecules (calcium, phosphorous, glucose, sodium, potassium, albumin, total protein, globulin, alkaline phosphatase, alanine transferase, amylase, total bilirubin, blood urea nitrogen and creatinine). Data points represent individual animals and line represents the average on a given day. (TIF) [file ppat.1009705.s005.tif]

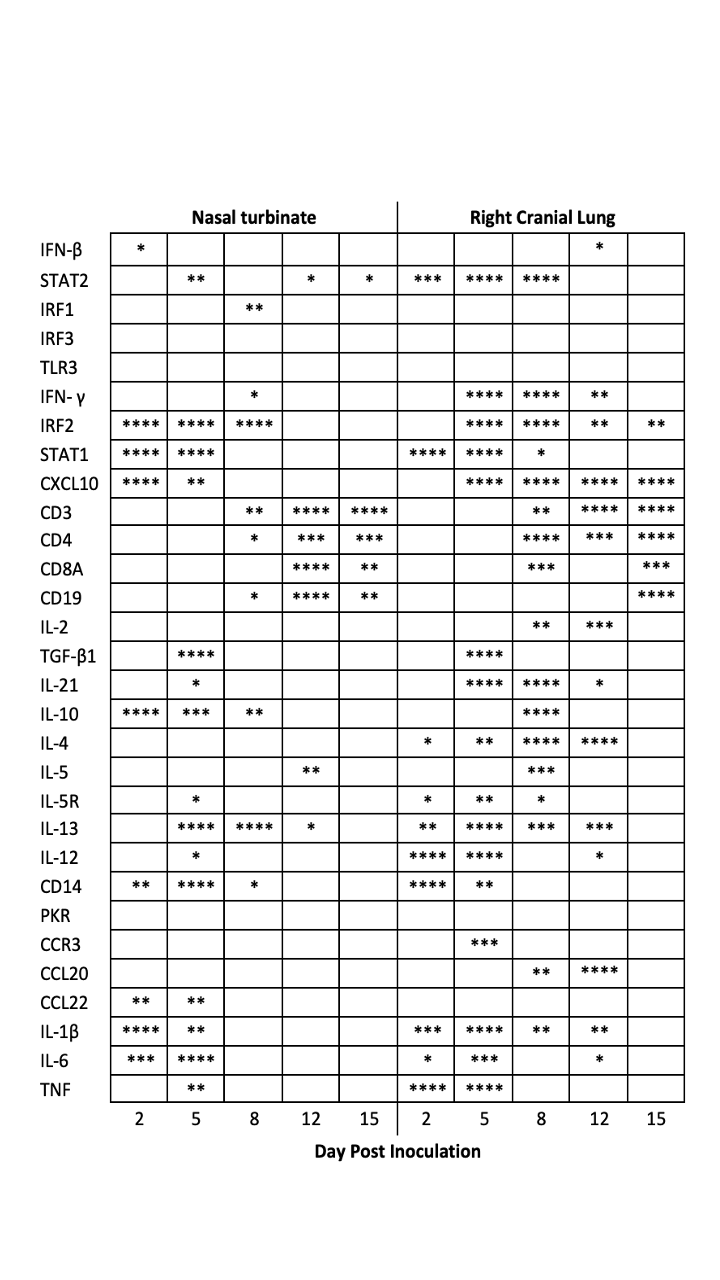

Supplement: S1 Table — qRT-PCR was performed on RNA extracted from nasal turbinate and right cranial lung tissue from SARS-CoV-2 inoculated hamsters. Fold-change was calculated via ΔΔCt against baseline (Day 0) with BACT as the housekeeping gene (Fig 5). ANOVA was used to calculate statistical significance by comparing hamsters on the days post inoculation to baseline (day 0). No asterisk indicates a p-value>0.05; *indicates a p-value < 0.05; **indicates a p-value <0.005; *** indicates a p-value <0.0005; **** indicates a p-value <0.0001. N is 3 for all timepoints. (TIFF) [file ppat.1009705.s006.tiff]

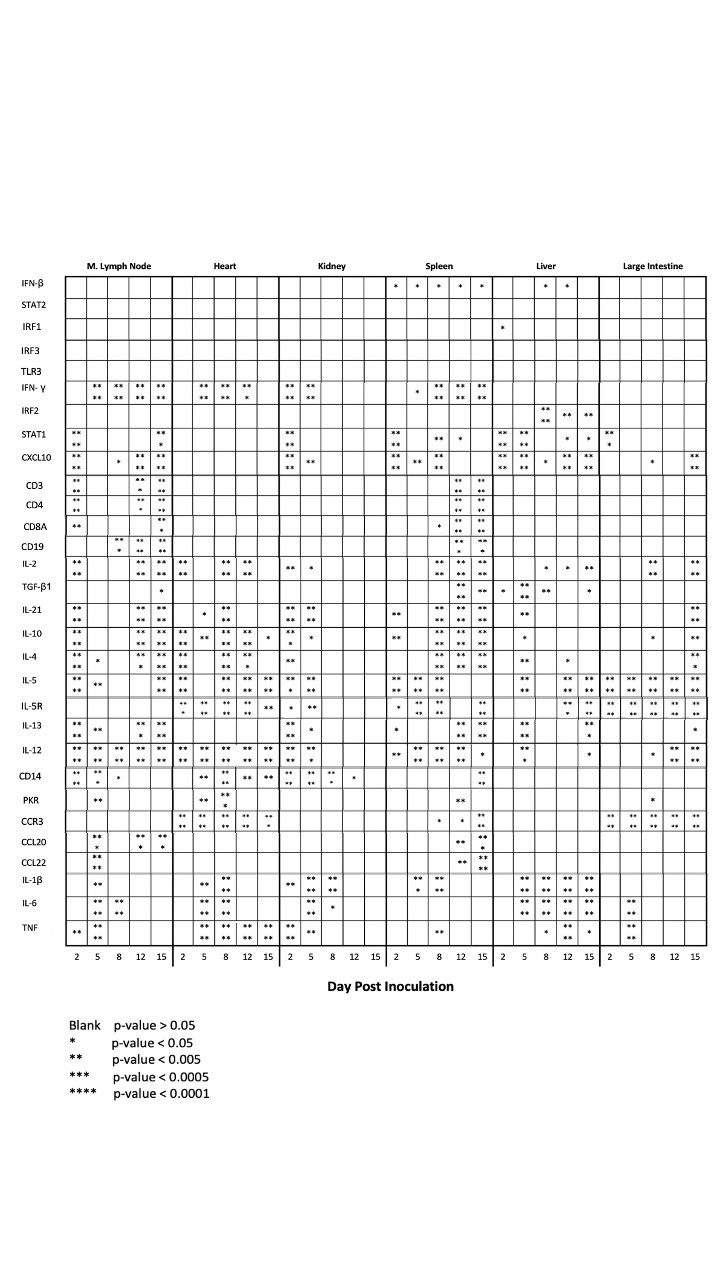

Supplement: S2 Table — qRT-PCR was performed on RNA extracted from mediastinal (M) lymph node, heart, kidney, spleen, liver, and large intestine from SARS-CoV-2 inoculated hamsters. Fold-change was calculated via ΔΔCt against baseline (Day 0) with BACT as the housekeeping gene (Fig 6). ANOVA was used to calculate statistical significance by comparing hamsters on the days post inoculation to baseline (day 0). No asterisk indicates a p-value>0.05; *indicates a p-value < 0.05; **indicates a p-value <0.005; *** indicates a p-value <0.0005; **** indicates a p-value <0.0001. N is 3 for all timepoints. (TIFF) [file ppat.1009705.s007.tiff]
